# Prevalence of early repolarization syndrome and long-term clinical outcome in patients with the diagnosis of idiopathic ventricular fibrillation

**DOI:** 10.1007/s00380-018-1273-7

**Published:** 2018-10-04

**Authors:** Daniel Dalos, Lukas Fiedler, Jovana Radojevic, Michael Sponder, Wolfgang Dichtl, Christoph Schukro

**Affiliations:** 1 Department of Internal Medicine II, Division of Cardiology, Medical University of Vienna, Waehringer Guertel 18-20, 1090 Vienna, Austria; 2 Department of Internal Medicine, Division of Cardiology, Landesklinikum Thermenregion Moedling, Mödling, Austria; 3 Department of Internal Medicine, Division of Cardiology, Medical University of Innsbruck, Innsbruck, Austria

**Keywords:** Idiopathic ventricular fibrillation, Early repolarization, Prevalence, Outcome

## Abstract

Idiopathic ventricular fibrillation (IVF) is diagnosed in up to 14% of sudden cardiac death (SCD) survivors. Early repolarization syndrome (ERS) in patients with ventricular tachyarrhythmia is characterized by an elevated J-point in inferior and/or antero-lateral leads. Our objectives were to determine the prevalence of ERS in IVF patients, and to evaluate potential differences in clinical outcome. Out of 3,552 implantable cardioverter defibrillator (ICD) carriers, 758 SCD survivors were retrospectively identified from the databases of the Medical Universities of Vienna and Innsbruck within the last three decades. Early repolarization pattern (ERP) was classified either as "notching" or "slurring". Endpoints were defined as appropriate ICD therapies for ventricular tachyarrhythmia, either anti-tachycardia pacing or shock, and all-cause mortality. After exclusion of recognized reasons for SCD, 50 patients were assigned to the diagnosis of IVF (6.6%). An ERP was identified in 10 patients, most of them with notching (*n* = 8). After a mean follow-up of 11.2 ± 6.7 years (539.3 patient years), appropriate ICD therapies were found in 50% of ERS and 43% of IVF patients without ERP (*p* = 0.732). In ERS patients, all ICD therapies were found in patients with notching pattern. Similarly, incidence of inappropriate ICD therapies, and all-cause mortality was comparable (30% vs. 23%, *p* = 0.707; 10% vs. 5%, *p* = 0.496, respectively). In 758 SCD survivors, we found a low prevalence of IVF and ERS. Similar event rates were reported concerning all-cause mortality and ICD therapies for ventricular tachyarrhythmia after long-term follow-up in this cohort.

## Introduction

Sudden cardiac death (SCD) is caused by malignant arrhythmia, predominantly ventricular fibrillation (VF). The highest susceptibility is reported in patients with structural heart disease [1], but also genetic mutations in ion channel proteins may trigger life-threatening arrhythmia [2]. In up to 14% of patients suffering from VF no underlying mechanism can be identified, which has been previously described as *idiopathic ventricular fibrillation* (IVF) [3]. Several working groups tried to determine risk factors for the occurrence of IVF, but no common cause for all IVF could be determined yet [4, 5].

Early repolarization patterns (ERP) were firstly depicted by Grant et al. in 1951 [6] and were initially related to a benign prognosis in a healthy population [7, 8]. However, various authors were able to demonstrate the clinical significance of ERP with an enhanced vulnerability to ventricular arrhythmias [9–14]. Over the last decades, knowledge increased concerning ERP in patients with Brugada syndrome [15] and acute coronary syndromes [16–18], but nevertheless there is still little information regarding ERP in patients with the diagnosis of IVF [9, 19].

This retrospective, multi-center analysis sought to investigate the prevalence of ERP in IVF and to assess potential differences in clinical outcome between patients with early repolarization syndrome (ERS; IVF_ER+) and IVF patients without ERP (IVF_ER-).

## Methods

### Definitions

In accordance with previously performed studies, ERP was defined as an elevated J-point ≥ 0.1 mV in ≥ 2 leads from the same regional territory of the 12-lead electrocardiogram [13, 14]. J-point elevation had to occur either as "notching" or "slurring" in inferior (II, III, aVF) or antero-lateral leads (I, aVL, V4-V6), or both.

*IVF* was defined as the absence of any channelopathy, ischemic, or structural cardiac abnormality as the underlying cause for ventricular fibrillation.

*ERS* was defined as SCD due to IVF *and* the appearance of the above described ECG patterns.

### Patient population

Patients were recruited post hoc from computed databases of two university hospital-centers. The study was approved by the institutional ethics committee and all patients gave their written informed consent.

Out of 3,552 implantable cardioverter defibrillator (ICD) carriers, 758 *sudden cardiac death survivors* were retrospectively identified over the last three decades.

After successful resuscitation, all patients underwent physical examination, 12-lead electrocardiogram (ECG; standard filter setting: 25 mm/s, 10 mm/mV, 40 Hz), transthoracic echocardiography and coronary angiography including adenosine- or nitroglycerin-testing to rule out vasospastic components. Based on the recent definition of 2016 and according to the consensus statement of 2013 [20, 21], after exclusion of significant coronary artery disease (stenosis ≥ 70%), cardiomyopathy (left ventricular ejection fraction ≤ 55% or right ventricular ejection fraction ≤ 50%) and significant valvular disease the following procedures have been performed in all patients: ajmaline-test to unmask Brugada syndrome and exercise ECG in order to exclude catecholaminergic polymorphic ventricular tachycardia. In addition, all ECGs were reviewed for long-QT or short-QT syndrome.

Starting from the year 2008, cardiac magnetic resonance imaging was performed in all patients, whenever there was no contraindication (e.g. metallic implants, claustrophobia, and chronic kidney disease with glomerular filtration rate < 30 ml/min.). Patients with positive late gadolinium enhancement were excluded.

**Fig. 1:**
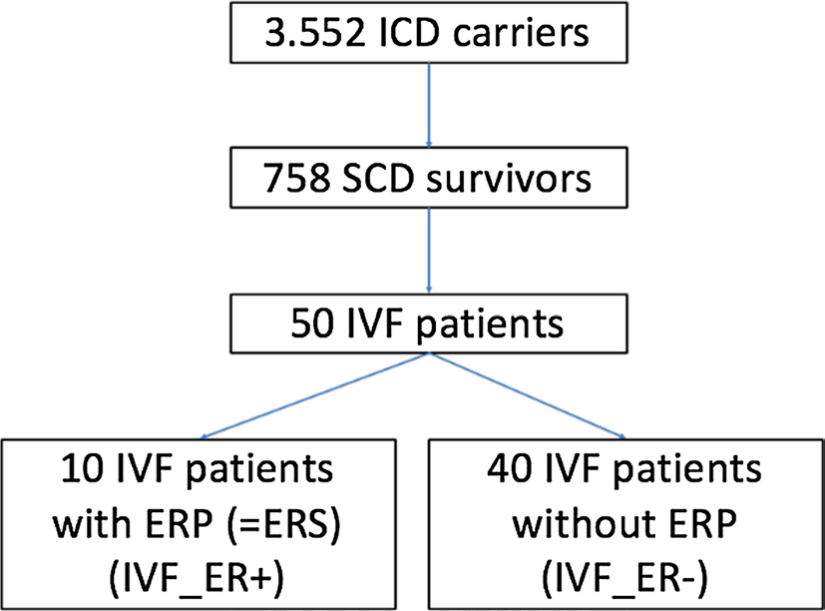
Patient selection. *ERP* early repolarization pattern, *ERS* early repolarization syndrome, *ICD* implantable cardioverter defibrillator, *IVF* idiopathic ventricular fibrillation, *SCD* sudden cardiac death

Finally, the diagnosis *idiopathic ventricular fibrillation* was established in 50 patients (Fig. 1).

Follow-up procedures, comprising brief physical examination, 12-lead ECG and device query, were performed as clinical routine by board certified cardiologists in ICD outpatient clinics every 6 months, as well as in case of symptomatic ICD therapies.

### Endpoints

Clinical endpoints were defined as appropriate ICD therapies, inappropriate ICD therapies and all-cause mortality. ICD therapies were defined either as shock delivery or anti-tachycardia pacing (ATP).

### Statistical analysis

Statistical analysis was performed using IBM SPSS Statistics version 21.0 (IBM, Armonk, NY). Data are expressed as frequencies or percentages for dichotomous variables and as mean ± standard deviation for continuous variables. Comparisons between groups were made using the Chi-square or Fisher's exact test for categorical variables, and the Student *t* test or Mann–Whitney *U* test for continuous variables, as appropriate. Two-sided *p* values < 0.05 were considered to indicate statistical significance.

## Results

Out of 1.88 million inhabitants of Vienna and 0.13 million inhabitants of Innsbruck (2.01 million in total), the prevalence of SCD survivors with subsequently implanted ICD was 38/100,000. Out of 50 patients with IVF, an ERP was identified in 10 patients (Fig. 1). Thus, prevalence of ERS in the overall population was 0.5/100,000 and 20% of all IVF patients revealed ERS (IVF_ER+). Mean age in IVF-ER+ patients was 58 ± 15 years, compared to 53 ± 15 years in IVF_ER- patients (*p* = 0.327), and most of them were male (70% vs. 73%, *p* = 1.000). All baseline characteristics are shown in Table 1 and were comparable between both groups except for amiodarone intake, which was more common in IVF_ER+ patients (30% vs. 5%, *p* = 0.048). Exactly half of the patients had single and dual chamber ICD devices.

**Table 1:** Baseline characteristics

	IVF_ER+ (*n* = 10)	IVF_ER- (*n* = 40)	*p *value
Age, years	58 ± 15	53 ± 15	0.327
Male sex, *n* (%)	7 (70)	29 (73)	1.000
Arterial hypertension, *n* (%)	5 (50)	16 (40)	0.723
Hyperlipidemia, *n* (%)	5 (50)	10 (25)	0.143
Atrial fibrillation, *n* (%)	3 (30)	7 (18)	0.397
Diabetes mellitus, *n* (%)	0	5 (13)	0.569
Family history of SCD, *n* (%)	0	1 (3)	1.000
Betablockers, *n* (%)	8 (80)	21 (53)	0.160
Amiodarone, *n* (%)	3 (30)	2 (5)	0.048
Sotalol, *n* (%)	0	1 (3)	1.000
Class IV antiarrhythmics, *n* (%)	0	2 (5)	1.000

*ER* early repolarization, *IVF* idiopathic ventricular fibrillation, *SCD* sudden cardiac death

Notching sign (*n* = 8) occurred in inferior leads in seven patients, was found in inferior *and* antero-lateral leads in three patients and in four patients in the antero-lateral leads. Slurring (*n* = 2) was found in one patient in the inferior leads, as well as in one patient in the antero-lateral leads only. Early repolarization pattern characteristics are presented in Table 2. Of note, in all patients with ERP, the typical pattern could be found in at least one, but not in each documented 12-lead ECG.

**Table 2:** Early repolarization pattern characteristics

	Notching (*n* = 8)	Slurring (*n* = 2)	*p *value
Inferior, *n* (%)	7 (86)	1 (50)	0.378
Antero-lateral, *n* (%)	4 (50)	1 (50)	1.000
Both, *n* (%)	3 (38)	0	1.000
Amplitude ≥ 0.1mV, *n* (%)	5 (62)	2 (100)	1.000
Amplitude > 0.2mV, *n* (%)	3 (38)	0	1.000

**Fig. 2:**
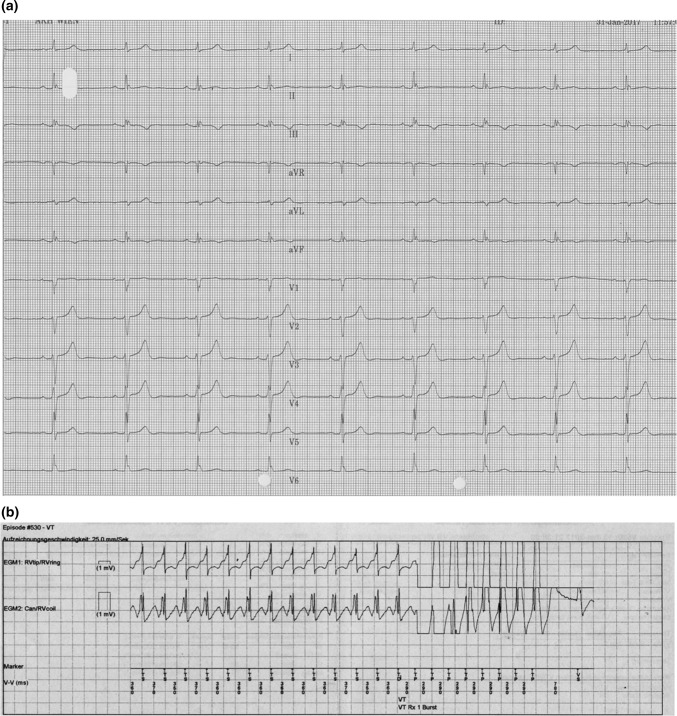
Anti-tachycardia pacing in a patient with early repolarization syndrome. **a** Early repolarization pattern in leads II, III, aVF and V6. **b** Intracardiac electrogramm with monomorphic ventricular tachycardia terminated by anti-tachycardia pacing

After a mean follow-up of 11.2 ± 6.7 years, corresponding to a total of 539.3 patient years, appropriate ICD therapies (ICD programming is shown in Table 3) were registered in nearly one half of patients in both groups (50% vs. 43%, *p* = 0.732), with comparable incidence of appropriate ATP or shock delivery, especially regarding the number of episodes and the total number of shocks (Table 4). Ventricular tachycardia was monomorphic in 7 of 12 patients in both groups (Fig. 2a, b). Additionally, we found comparable rates of inappropriate therapies (30% vs. 23%, *p* = 0.707). Main reasons for inappropriate shock delivery or ATP were sinus tachycardia, atrial fibrillation (AF), and lead fracture (Table 4).

**Table 3:** Standard programming of implantable cardioverter-defibrillator

	Slow VT zone	Fast VT zone	VF zone
Cycle length (ms)	375–300	300–250	< 250
Anti-tachycardia therapy	(1) Burst ATP (2) Ramp ATP	(1) Burst ATP (2) Ramp ATP (3) Shock	Shock (burst-before-shock if available)
ATP coupling interval (%)	80–85	78–81	Not applicable

*ATP* anti-tachycardia pacing, *VF* ventricular fibrillation, *VT* ventricular tachycardia

**Table 4:** Clinical endpoints

	IVF_ER+ (*n* = 10)	IVF_ER- (*n* = 40)	*p *value
Appropriate therapy, *n* (%)	5 (50)	17 (43)	0.732
Appropriate shock, *n* (%)	5 (50)	13 (33)	0.463
Number of episodes, mean ± SD	4 ± 6	5 ± 6	0.387
Number of shocks, mean ± SD	11 ± 17	6 ± 7	0.703
Appropriate ATP, *n* (%)	3 (30)	9 (23)	0.686
Inappropriate therapy, *n* (%)	3 (30)	9 (23)	0.707
Inappropriate shock, *n* (%)	3 (30)	8 (20)	0.671
AF, *n* (%)	1 (10)	2 (5)	0.661
Sinus tachycardia, *n* (%)	0	6 (60)	0.061
Lead fracture, *n* (%)	1 (33)	1 (3)	0.491
EMI, *n* (%)	1 (10)	0	0.333
Inappropriate ATP, *n* (%)	0	1 (3)	1.000
Appropr. *and* inappr. therapy, *n* (%)	3 (30)	4 (10)	0.133
Appropriate therapy only, *n* (%)	2 (20)	12 (30)	0.704
Inappropriate therapy only, *n* (%)	0	5 (13)	0.569
All-cause mortality, *n* (%)	1 (10)	2 (5)	0.496

*AF* atrial fibrillation, *ATP* anti tachycardia pacing, *EMI* electro magnetic interference, *ER* early repolarization, *IVF* idiopathic ventricular fibrillation

Although IVF_ER+ patients suffered from more appropriate *and* inappropriate ICD therapies, inappropriate therapies solely were more common in IVF_ER-, but both distinctions did not reach statistical significance (30% vs. 10%, *p* = 0.133; 0% vs. 13%, *p* = 0.569, respectively; Table 4).

After thorough assessment of the different appropriate and inappropriate ICD therapies, we actually found no case of accelerated ventricular tachyarrhythmia. Finally, all-cause mortality was low in both groups (Table 4).

## Discussion

This retrospective, multi-center analysis shows a low prevalence of IVF in a large ICD patient cohort of two high-volume centers. In this small patient cohort, ERP was identified in one fifth of all IVF patients, with comparable all-cause mortality and in-/appropriate ICD therapies, after a mean follow-up duration of more than one decade.

Prevalence of ERS in our overall population was 0.5/100,000, whereas in the recent literature, this prevalence varied from 3 to 24% [22]. Previous studies described a higher prevalence of early repolarization pattern in young individuals, especially those with vagotonia, males, African Americans and athletes [23, 24]. Some of these findings are in line with the characterization of our IVF-ER+ cohort (mean age 58 ± 15 years, 70% male). It's worth noting, that in prior studies the terms of ERP and ERS were used synonymously. Most patients with the typical ECG patterns did not suffer from SCD and therefore did not fulfill the criteria of this syndrome.

In a recent meta-analysis, Cheng et al. investigated 16 studies with 334,524 patients and found that the presence of ERP is associated with a significant risk for SCD, cardiac death and death from any cause in the general population [25]. Furthermore, the same group was able to point out the prognostic relevance of ERP in patients with structural heart disease [26]. These results are in accordance with the ones observed by Holmström et al., who performed autopsies of 275 non-ischemic SCD victims, and suggested infero-lateral ERP to be associated with an increased risk for non-ischemic SCD. Prevalence of ERP was significantly higher in the SCD group, compared to the general population cohort with 10,864 subjects (21% vs. 5%, *p* < 0.001) [27].

Nevertheless, there is only little evidence concerning ERP in SCD patients with IVF. In 2008, Haïssaguerre et al. published multi-center data from 206 SCD patients who were resuscitated due to idiopathic ventricular fibrillation, showing an ERP prevalence of 31% compared to 5% in 412 control subjects without heart disease (*p* < 0.001) [9]. This finding is consistent with the results of Rosso et al., who found J-point elevation more common in IVF patients than in matched controlled subjects (42% vs. 13%, *p* = 0.001) [12]. Similarly, we found a prevalence of 20%, which might be lower due to our smaller patient cohort. Siebermair et al. found an ERP prevalence of 37% among 35 IVF patients and identified ERP as an independent predictor of arrhythmia recurrence [19].

However, to the best of our knowledge, our study is the first one to investigate clinical and technical characteristics in IVF patients with and without ERP, although observations were made in a small patient population. Incidence of appropriate ICD therapies due to ventricular arrhythmia was similar between these groups. Remarkably, monomorphic ventricular tachycardia (VT) was documented and terminated by appropriate ATP in both groups, irrespectively of the presence of an ERP. This finding could suggest that monomorphic VT might be the initial arrhythmia before degenerating in ventricular fibrillation. But as no “macroscopic” substrate for monomorphic VT has been found in our cohort, we have to suspect an electrophysiological substrate. This theory was recently supported by Haïssaguerre et al. [28]. In this study, predominantly epicardial focal electrogram abnormalities were documented in almost two thirds of the investigated patients with IVF. And in most of the remaining patients, the authors observed a high incidence of Purkinje triggers as a potential arrhythmia substrate in this collective.

Trenkwalder et al. [29] concluded that the presence of ERP is not associated with an increased occurrence of ventricular or supraventricular arrhythmia, although the study was performed in a population-based cohort and no IVF patients have been assessed. The group of Haïssaguerre showed a significantly higher incidence of recurrent ventricular fibrillation in patients with ERP [9]. This is in contrast to our findings, where not only the number of shock episodes, but also the total number of shocks was comparable between IVF_ER+ and IVF_ER- patients during a mean follow-up of 11 years.

Furthermore, we found comparable rates of inappropriate ICD therapies. In the cohort of Siebermair et al., 40% of all IVF patients experienced inappropriate ICD shocks, mainly due to rapidly conducted AF [19]. In our cohort, we could not find differences in prevalence of AF between IVF_ER+ and IVF_ER- patients. Moreover, atrial fibrillation was a rare cause for inappropriate shock delivery, supporting the theory of missing association between ERP and AF from Junttila et al. [10].

In our total cohort of IVF patients, we did not find any case of accelerated ventricular tachyarrhythmia after appropriate and inappropriate ICD therapies. This is in line with a previously published finding from our institution, where acceleration of ventricular tachyarrhythmia—mainly triggered by appropriate ATP—has shown to be a complication that predominantly affects patients with structural heart disease, mainly with reduced left ventricular systolic function [30].

Finally, ERP were found in at least one, but not in each documented 12-lead ECG in patients with IVF_ER+. This finding is consistent with our previous report on another channelopathy, where diagnostic type I Brugada pattern was not recorded in every ECG [31]. Additionally, Aizawa et al. were able to show a dynamicity of J waves in IVF patients and revealed a pause-dependent augmentation after R-R interval prolongation [32]. Therefore, ERP might occur more frequently and may be under-diagnosed in clinical practice, especially in the subset of IVF patients. This fact supports the need of larger prospective trials with focus on frequent ECG recordings.

## Limitations

Given the retrospective, observational nature of our patient cohort over a time frame of three decades, some diagnostic modalities, e.g. cardiac magnetic resonance imaging, were not performed systematically. This might have led to a specific number of patients with under-diagnosed structural abnormalities that do not fulfill criteria of IVF.

Furthermore, 12-lead ECG recordings were collected from different points of time before and after ICD implantation. Because all retrospectively analyzed ECGs after successful resuscitation were performed at the emergency department, potential ERP at first medical contact could not be assessed, which might result in lower prevalence rates.

## Conclusion

In conclusion, we report a relatively low prevalence of IVF out of 758 SCD survivors in two high-volume centers. One fifth of all IVF patients showed typical patterns of early repolarization, with similar event rates regarding all-cause mortality and ICD therapies for ventricular tachyarrhythmia after long-term follow-up compared to IVF patients without ERP. Nevertheless, the relatively high rates of arrhythmia recurrence support the necessity of ICD implantation and ensure prospective studies, in order to determine independent predictors of outcome in this specific population.
